# Workload increases nurses' quiet quitting, turnover intention, and job burnout: evidence from Greece

**DOI:** 10.3934/publichealth.2025004

**Published:** 2025-01-07

**Authors:** Petros Galanis, Ioannis Moisoglou, Aglaia Katsiroumpa, Parisis Gallos, Maria Kalogeropoulou, Evangelia Meimeti, Irene Vraka

**Affiliations:** 1 Clinical Epidemiology Laboratory, Faculty of Nursing, National and Kapodistrian University of Athens, Athens, Greece; 2 Faculty of Nursing, University of Thessaly, Larissa, Greece; 3 Third Regional Health Authority of Macedonia, Thessaloniki, Greece; 4 Department of Radiology, P & A Kyriakou Children's Hospital, Athens, Greece

**Keywords:** workload, quiet quitting, turnover intention, job burnout, nurses, COVID-19

## Abstract

**Introduction:**

High workloads among nurses affect critical workplace outcomes, such as turnover intention, job burnout, and job satisfaction. However, there are no studies that measure the relationships between workload and these variables in the post-COVID-19 era.

**Objective:**

To evaluate the effect of workload on quiet quitting, turnover intention, and job burnout.

**Methods:**

We conducted a cross-sectional study using a sample of nurses in Greece. The NASA task load index was used to measure workloads among nurses. Also, we used valid scales to measure quiet quitting (quiet quitting scale), job burnout (single item burnout measure), and turnover intention (a six-point Likert scale).

**Results:**

The mean workload score was 80.7, indicating high workloads in our sample. Moreover, most of the nurses belonged to the group of quiet quitters (74.3%). About half of the nurses reported a high level of turnover intention (50.2%). After controlling for confounders, data analysis showed that higher workloads were associated with higher levels of quiet quitting [beta = 0.009, 95% confidence interval (CI) = 0.006 to 0.012, p-value < 0.001], turnover intention (odds ratio = 1.046, 95% CI = 1.035 to 1.056, p-value < 0.001), and job burnout (beta = 0.072, 95% CI = 0.065 to 0.079, p-value < 0.001).

**Conclusion:**

We found that workload was associated with quiet quitting, turnover intention, and job burnout in nurses. Thus, appropriate interventions should be applied to reduce nursing workloads to improve productivity and the healthcare provided to patients.

## 1. Introduction

Modern healthcare systems face major challenges, such as the increase in the proportion of elderly people in the population, the growing demand for healthcare services, the large number of elderly people with chronic deceases and comorbidities, and the constant demand to improve the quality and safety of health care services [1],[2]. All of the above create a complex working context leading to an increase in nurses' workloads, as demands increase for both direct and indirect nursing care.

Nursing workload is defined as “*the totality of the need for nursing time from all work that must be carried out over a defined period of time*” [3]. However, apart from the external factors that can contribute to the increase in nursing workloads, such as clinical characteristics and comorbidities of patients, inadequate supply of nurses, and the organization and functioning of the hospital, there are a number of other elements linked to the organizational context of the nurses' working environments that are in responsible for the workload increase. In the midst of the COVID-19 outbreak, the massive admissions of patients with severe disease, the need for COVID-19 ward and ICU beds, and the isolation of patients from their relatives, created a particularly high workload for nurses [4]. Nursing understaffing is one of the most significant organizational elements affecting nurses' working environments. Nursing understaffing is not a modern problem of health systems, but a long-standing failure of health care organizations to ensure adequate nursing staffing in different countries and settings [5]–[7]. Forecasts for the nursing workforce are bleak, with shortages estimated at millions of nurses [8],[9]. The COVID-19 pandemic exacerbated the situation and caused thousands of nurses to abandon their careers. Another large number of nurses declared their intention to leave the profession in the upcoming years [10]. The increased nursing workload associated with understaffing negatively affects the quality and safety of the nursing care provided [11]–[14]. Nurses who experience high workloads are much more likely to develop burnout, to be absent from work, and to commit errors when caring for their patients [15]. Research has also been carried out in Greece to examine the workload of nursing personnel. The results indicate that workload decreases job satisfaction and increases occupational stress [16],[17]. A study conducted with mental health nurses revealed a substantial negative relationship between workload satisfaction and burnout [18].

During the COVID-19 pandemic, employees in the business sector adopted a working behavior called quiet quitting. Employees who choose quiet quitting do not leave their position or profession, but remain in it, offering what is absolutely necessary to avoid getting fired. They do not show up early, avoid working overtime and do not express new ideas. Their aim is to reduce the burden of their work and balance their work and family life [19]. The initial study that brought this important issue to the forefront was in the US business sector, where over 50% of workers were observed to favor the quiet quitting [20]. The development of a reliable and valid tool has enabled researchers to investigate this phenomenon in the health sector, although this tool can be used in all business sectors [21]. Early studies in the healthcare sector showed the extent of the phenomenon, with nurses compared to other healthcare professionals choosing quiet quitting at a rate of over 60% [22]. Burnout and bullying have been identified as contributing factors to the occurrence of quiet quitting, while emotional intelligence and moral resilience reduce the likelihood of this work-related behavior [23]–[26].

According to Maslach et al. “*Burnout is a prolonged response to chronic emotional and interpersonal stressors on the job, and is defined by the three dimensions of exhaustion, cynicism, and inefficacy*” [27]. Burnout has a very high impact on healthcare professionals, with nurses having the highest rates among them [28],[29]. When nurses are employed in a setting where there is a lack of personnel, lack of available material resources, heavy workload, lack of support for nurses from their organization, poor working relationships with medical staff, and the absence of nurses' participation in hospital issues, the chances of developing burnout increase [30]–[32]. Burnout among nurses is a factor that reduces their organizational commitment and productivity, as well as the quality and safety of the care they provide [33],[34]. In a recent study, burnout emerged as one of the main factors contributing to nurses terminating their employment in the healthcare sector [35].

When nurses find that the organizations they work for are failing to provide them with a healthy working environment, they often declare their turnover intention. The decision made voluntarily by a nurse to change the job position either within the same organization or to a different healthcare institution, or ultimately resigning the profession and seeking a new career path, is referred to as nurses' turnover [36]. Over time, a significant portion of nurses indicate that they intend to leave their jobs [37]. Among the most important predictors of turnover intention include high workload, lack of organizational support, job dissatisfaction, and job burnout [37]–[39]. While the term “turnover intention” describes a nurse's propensity to resign from an organization in the short term, research indicates that a sizable percentage of nurses actually choose to leave [40]. High turnover intention rates are thus a red flag that should spur nurse managers and stakeholders to improve work conditions for nurses.

Therefore, our purpose was to examine the effect of workload on quiet quitting, turnover intention, and job burnout in nurses. As far as we are aware, this is the first study that examined the impact of workload on levels of quiet quitting in nurses. Additionally, we investigated the impact of workload on job burnout, and turnover intention for first time in the post COVID-19 era.

## 2. Materials and methods

### 2.1. Study design

In October 2023, a cross-sectional survey was carried out in Greece. Nurses employed in a healthcare setting for a minimum of two years, comprised our sample. Our data was gathered via an online survey. In particular, we created an online version of the study questionnaire and disseminated it through social media (e.g., Facebook, Instagram, LinkedIn, and Viber). Thus, convenience sampling method was employed to approach our sample. We calculated a minimum sample size of nurses as 652 by applying the following parameters: (a) a low effect size (f^2^ = 0.02) between workload and quiet quitting, turnover intention, and job burnout; (b) five confounders; (c) one predictor; (d) 95% level of confidence; and (e) 5% margin of error. We employed the G*Power software (version 3.1.9.2) to calculate the sample size of nurses. Our study protocol was approved by the Ethics Committee of the Faculty of Nursing, National and Kapodistrian University of Athens (approval number; 459, September 2023). Moreover, our study was carried out in accordance with the Declaration of Helsinki [41].

### 2.2. Measurements

Demographic data, including gender, age, understaffed department, shift work, and clinical experience, were recorded. Nurses were asked to self-report whether they believe that their department was understaffed or not.

The NASA task load index (NASA-TLX) was used to measure workload among our nurses [42]. The NASA-TLX includes six items assessing workload: mental demand, physical demand, temporal demand, performance, effort, and frustration. By combining the six items, we create an overall workload score from 0 to 100. Higher values indicate higher level of workload. The valid Greek version of the NASA-TLX was used [43]. Cronbach's α for the NASA-TLX was 0.742 in our study.

The quiet quitting scale (QQS) was used to measure levels of quiet quitting in our sample [21]. The QQS comprises nine items with answers in a 5-point Likert scale from 1 (totally disagree) to 5 (totally agree). Total score ranges from 1 to 5. Higher values indicate higher level of quiet quitting. Developers of the scale suggest a cut-off point score (equals to 2.06) to divide employees in quiet quitters and non-quiet quitters [44]. A study with nurses in Greece showed very good psychometric properties for the QQS [22]. Cronbach's α for the QQS was 0.852 in our study.

The single-item burnout measure was used to estimate job burnout in our sample [45]. This item takes value from 0 to 10. According to the single-item burnout measure, nurses were asked to rate their current level of burnout. The single-item burnout measure is based on the Maslach burnout inventory and the developers of the measure examined its concurrent validity by using the Maslach burnout inventory. Higher values indicate higher level of job burnout. The valid Greek version of the scale was used [46].

A valid 6-point Likert scale was used to measure turnover intention among our nurses [47]. It is a single item scale to measure turnover intention. In particular, we asked nurses “How often have you seriously considered leaving your current job?” and answers were on a scale from 1 (rarely) to 6 (extremely often). A suggested cut-off point of four divides participants in two groups: low or high level of turnover intention among workers.

### 2.3. Statistical analysis

Frequencies and percentages were used to present categorical variables. In case of continuous variables, mean, standard deviation, median, minimum value, and maximum value were used. Linear regression analysis was applied to examine the effect of workload on quiet quitting and job burnout. Additionally, logistic regression analysis was conducted to examine the effect of workload on turnover intention since turnover intention was a dichotomous variable. Univariate and multivariable regression models were constructed to examine the impact of workload. In case of multivariable regression models, the following confounders were eliminated: gender, age, understaffed department, shift work, and work experience. In general, females, older workers, workers in understaffed departments, shift workers, and workers with increased number of work experience higher levels of quiet quitting, turnover intention, and job burnout. Coefficients beta, 95% confidence intervals (CI), p-values, and R^2^ are presented for linear regression models. Odds ratios (OR), 95% confidence intervals, p-values, and R^2^ are presented for logistic regression models. P-values less than 0.05 were considered as statistically significant. IBM SPSS 21.0 (IBM Corp. Released 2012. The IBM SPSS Statistics for Windows, Version 21.0. Armonk, NY: IBM Corp.) was used for statistical analysis.

## 3. Results

### 3.1. Participant characteristics

The mean age of the 1092 nurses in our sample was 42.2 years. The majority of nurses were females (90.3%) working in understaffed departments (80.9%). Seven out of ten nurses have been shift workers. Average years of employment were 17.1. Detailed demographic characteristics are displayed in Table 1.

**Table 1.** Sample characteristics (N = 1092).

Characteristics	N	%	Mean (standard deviation)
Gender			
Females	986	90.3	
Males	106	9.7	
Understaffed department			
No	209	19.1	
Yes	883	80.9	
Shift work			
No	353	32.3	
Yes	739	67.7	
Age (years)			42.1 (9.6)
Clinical experience (years)			17.1 (10.1)

### 3.2. Study scales

The mean score for workload was 80.7 (SD = 13.7, range 0–100), 2.6 (SD = 0.7, range 1–5) for quiet quitting, and 7.5 (SD = 1.9, range 0–10) for job burnout among nurses. In our sample, 50.2% (n = 548) of nurses reported a high level of turnover intention, while 49.8% (n = 544) reported a low level of turnover intention.

### 3.3. Impact of workload on quiet quitting, turnover intention, and job burnout

We revealed a positive relationship between workload and quiet quitting, turnover intention, and job burnout. After controlling for confounders (i.e., gender, age, understaffed department, shift work, and clinical experience), we observed that a greater workload was linked to a higher level of quiet quitting (unstandardized beta = 0.009, 95% confidence interval = 0.006 to 0.012, standardized beta = 0.169, p-value < 0.001) (Table 2). Additionally, higher level of workload was linked to an increased probability of turnover intention (odds ratio = 1.046, 95% confidence interval = 1.035 to 1.056, p-value < 0.001) (Table 3). Moreover, multivariable linear regression model indicated a positive relationship among workload and job burnout (unstandardized beta = 0.072, 95% confidence interval = 0.065 to 0.079, standardized beta = 0.517, p-value < 0.001) (Table 4). Tables 2–4 show the full multivariable models including the following confounding variables: gender, age, understaffed department, shift work, and work experience.

**Table 2.** Linear regression analysis with quiet quitting as the dependent variable (N = 1092).

**Independent variable**	**Univariate model**			**Multivariable model^a^**		
	**Unstandardized coefficient beta (standardized)**	**95% CI for beta**	**P-value**	**Unstandardized coefficient beta (standardized)**	**95% CI for beta**	**P-value**
Workload	0.008 (0.142)	0.004 to 0.011	<0.001	0.009 (0.169)	0.006 to 0.012	<0.001

Note: ^a^R^2^ = 4.6%, p-value for analysis of variance < 0.001; CI: confidence interval.

**Table 3.** Logistic regression analysis with turnover intention as the dependent variable (N = 1092), (reference category: low level of turnover intention).

**Independent variable**	**Univariate model**			**Multivariable model^a^**		
	**Odds ratio**	**95% CI for odds ratio**	**P-value**	**Odds ratio**	**95% CI for odds ratio**	**P-value**
Workload	1.047	1.037 to 1.057	<0.001	1.046	1.035 to 1.056	<0.001

Note: ^a^R^2^ = 11.4%, p-value for analysis of variance < 0.001; CI: confidence interval.

**Table 4.** Linear regression analysis with job burnout as the dependent variable (N = 1092).

**Independent variable**	**Univariate model**			**Multivariable model^a^**		
	**Unstandardized coefficient beta (standardized)**	**95% CI for beta**	**P-value**	**Unstandardized coefficient beta (standardized)**	**95% CI for beta**	**P-value**
Workload	0.078 (0.555)	0.071 to 0.084	<0.001	0.072 (0.517)	0.065 to 0.079	<0.001

Note: ^a^R^2^ = 35.0%, p-value for analysis of variance < 0.001; CI: confidence interval.

## 4. Discussion

For the first time, we looked into how workload affects quiet quitting. Additionally, we evaluated the relation between workload and job burnout and turnover intention in nurses. We examined these relationships after the elimination of several confounders. We found that higher workload was associated with higher levels of quiet quitting, turnover intention, and job burnout in nurses. Our results agree with those of previous research in the field [31],[48]. Regarding workload and quiet quitting, the present study is the first in the literature to indicate the existence of a relationship between these two variables. The high workload resulting from understaffing of hospitals and the increased nurse-to-patient ratio have been found in a large European study in 6 countries to be linked with the occurrence of burnout in both physicians and nurses [49]. Within this European study, health professionals stated that improving staffing is the most effective intervention in improving their own well-being. Also, in a study conducted in US, burnout has been identified as the main factor driving nurses' decision to leave their position. Also, those who leave their position due to burnout report that their work environment is characterized by inadequate staffing [50]. Therefore, nurse understaffing appears to be an important variable affecting workload, which in turn could trigger burnout and leaving the job.

Considering the values of R^2^ for our multivariable models we found that the highest R^2^ (35.0%) referred to the relationship between workload and job burnout, and the lowest R^2^ (4.6%) referred to the relationship between workload and quiet quitting. Moreover, workload explained 11.4% of the dichotomous variable “turnover intention” in the multivariable logistic regression model. Therefore, workload explained job burnout and turnover intention more than quiet quitting. Since quit quitting is a new phenomenon among workers it is reasonable that predictors of this phenomenon are unknown and still under study. We examined one potential determinant of quiet quitting (i.e., workload), but several other variables may explain quiet quitting. On the other hand, workload explained a high percentage of job burnout and turnover intention since higher workloads result on higher levels of burnout and, thus, turnover intention. This finding is consistent with a similar finding in a study that included Greek mental health nurses, where the level of satisfaction with workload was identified as a predictor of burnout [18]. Moreover, in another Greek study, it was found that an augmented workload had a positive effect on burnout [51].

Nurse staffing levels and workload are tightly correlated to nurse's turnover [31],[52]. In turn, the turnover intention of nurses or even resign from their job, may exacerbate the already understaffed departments. The process of recruitment to fill vacancies is time consuming and costly [53], and this should be added to the time required for newly recruited nurses to fully undertake their duties. As nurse's turnover intention eventually leads to an actual decision to leave, and given the very high prevalence of turnover intention rates of up to 75% [54], turnover intention is a constant threat to nursing staffing. This creates a vicious cycle of understaffing, workload and turnover intention, trapping both nurses and organizations in a perpetually distressed and inefficient situation. Therefore, health service managers should seriously consider improving nursing staffing as a cost-effective practice in an effort to address nursing staff turnover.

Preliminary evidence suggests that more and more employees, including those in the health sector, are opting for quiet quitting as an attempt to self-protect themselves in challenging working conditions. On the one hand, we have the high workload of nurses, who frequently lack the time necessary to attend to their patients' needs [55],[56], and on the other hand we have nurses who choose quiet quitting, providing the bare minimum of efforts. This situation can lead to a reduction in the effectiveness of nurses, the deterioration of quality and patient safety, while at the same time it is an obstacle to any effort of innovation and improvement. Despite the fact that nurses are choosing quiet quitting in the face of challenging circumstances, this is insufficient to keep them in their current positions. In one study, where 60% of nurses chose quiet quitting, data analysis revealed that greater levels of quiet quitting increased turnover intention [57]. As, according to the findings of the present study, workload is a significant factor in quiet quitting accounting for 4.6% of quiet quitting in this sample, reducing the workload may modify their work behavior from choosing quiet quitting to motivating them to more efficient work behavior.

There were various limitations in our study. To collect our data, we used social media to administer an online survey. Furthermore, a convenience sample was used. Despite achieving the minimum sample size required our sample was not representative of Greek nurses, so we are unable to generalize our findings. Since the information regarding the demographic characteristics of nurses' population in Greece is unknown we cannot make direct comparisons with our sample. However, since we collected our data through social media we expected that our nurses would be younger than population of nurses in Greece. Additionally, it is probable that the percentage of females in our study would be an overestimation of the true percentage in the population of nurses since nine out of ten nurses were females. By using random and stratified samples of nurses in future research, this bias might be reduced. We measured workload, quiet quitting, turnover intention, and job burnout using valid instruments. Since these are self-reported instruments, our study is likely to have information bias. We eliminated several confounders in the relationship between workload and quiet quitting, turnover intention, and job burnout. However, further confounders can be eliminated in future studies. We are unable to draw conclusions about a link of causation between workload and quiet quitting, turnover, or job burnout because our study was cross-sectional. This bias might be reduced by longitudinal studies that track nurses' attitudes over time.

## 5. Conclusions

This study revealed the relationship between workload and quiet quitting, burnout and turnover intention among nurses. Although, the modern profile of patients is changing, making their care more demanding and increasing the workload of nurses, understaffing remains a major unresolved issue in healthcare services. Currently, the healthcare professional group with the highest rates of burnout, turnover, and quiet quitting is nurses, which leads to a deterioration in quality of care and nursing shortages. The increased workload is exacerbating the existing situation, making it imperative that health service administrations address staffing and workload issues. For the first time in the post-COVID-19 era, we looked into the effect of workload on quiet quitting in a sample of nurses. To further validate the results of our study, more research is required.

## Use of AI tools declaration

The authors declare they have not used Artificial Intelligence (AI) tools in the creation of this article.
